# RPN1 promotes the proliferation and invasion of breast cancer cells by activating the PI3K/AKT/mTOR signaling pathway

**DOI:** 10.1007/s12672-024-00875-8

**Published:** 2024-02-01

**Authors:** Wei-juan Shen, Yi Zhang

**Affiliations:** 1 Department of Breast surgery, Changzhou Wujin People’s Hospital (Wujin Hospital Affiliated with Jiangsu University), Changzhou, 213004 Jiangsu China; 2 Department of Breast surgery, The Wujin Clincal college of Xuzhou Medical University, Changzhou, 213004 Jiangsu China

**Keywords:** RPN1, Breast cancer, PI3K/AKT/mTOR, Migration, Invasion

## Abstract

Ribophorin I (RPN1), a part of an N-oligosaccharyl-transferase complex, plays a vital role in the development of multiple cancers. However, its biological role in breast cancer has not been completely clarified. The RPN1 expression level was measured in breast cancer tissues and breast cancer cell lines (MCF7) using RT-qPCR. After down-regulating RPN1 expression by shRNA, the effects of RPN1 on the proliferation, migration and invasion of MCF7 cells were examined. Mechanistically, we assessed the effect of RPN1 on the PI3K/ AKT/mTOR signaling pathway. We found that RPN1 level was up-regulated in breast cancer tissues and cells compared with adjacent non-tumor tissues or MCF10A cells. RPN1 knockdown induced apoptosis and attenuated the proliferation, migration, and invasion of MCF7 cells. Moreover, RPN1 knockdown lowered the levels of p-PI3K/PI3K, p-AKT/AKT, and p-mTOR/mTOR, which were rescued by 740Y-P, a PI3K activator. 740Y-P also reversed the effects of RPN1 knockdown on apoptosis, proliferation, migration, and invasion in MCF7 cells. Taken together, RPN1 promotes the proliferation, migration, and invasion of breast cancer cells via the PI3K/AKT/mTOR signaling pathway.

## Introduction

Breast cancer is one of the most common cancers in women worldwide and its incidence is increasing year by year [1, 2]. Despite advancements in routine screening methods and clinical treatment regimens, its patients frequently show metastasis [3, 4]. To date, breast cancer has no effective treatment options. Therefore, it is important to understand the mechanisms underlying the development of breast cancer. Breast cancer is a complex disease characterized by the uncontrolled growth and division of cells in the breast tissue [5]. It is influenced by a variety of factors, including genetics, environment, and individual lifestyle [5, 6]. The 5-year survival rate for patients diagnosed with metastatic breast cancer is only 15% [7]. There is an urgent need to understand the mechanisms of breast cancer initiation and progression and to develop new therapeutic targets. Therefore, revealing the underlying these mechanisms aids in the discovery of diagnostic biomarkers and new therapeutic targets for breast cancer.

A contributor to the failure of breast cancer treatment is that all breast cancer cells and their microenvironment capillary endothelial cells express asparagine linked glycoproteins with different structures, according to Banerje et al. Therefore, they tested a small biomolecule called Tunicamycin to block the catalytic activity of acetylglucosamine 1-phosphate transferase (GPT) in the protein glycosylation pathway of the endoplasmic reticulum (ER). The mechanistic details support that the unfolded protein response signal induced by ER stress is the culprit of the apoptosis of cancer cells and microvascular endothelial cells [8]. In addition, mucinous o-chain glycosylation changes are seen in more than 90% of breast cancers, and the mechanisms involved are increased/altered expression of glycosyltransferase and relocalisation to the ER of the enzyme responsible for adding the first sugar—acetyl-D-galactosamine [9]. It has been found that these changes can promote tumor growth and progression by regulating the microenvironment through glycan-sensing lectins expressed on immune cells, interactions with tumor surface receptors, and binding to selectins [9]. In order to identify new potential targets for therapeutic intervention in breast cancer, it is critical to comprehend the effects of ER and glycoproteins on tumor growth and progression.

Ribophorin I (RPN1) is a protein involved in protein synthesis and the formation of glycoproteins in ER [10, 11]. RPN1 acts as a receptor and regulator of protein translocation in the ER. It helps guide and anchor nascent proteins to the ER membrane, facilitating their proper folding and glycan modification. These results are essential for the production of functional proteins that are destined for secretion or reside in the cell membrane [12]. Emerging studies suggest that RPN1 may play a role in cancer progression [10]. Alterations in RPN1 expression or function have been observed in certain types of cancer, including breast cancer. Changes in RPN1 levels can affect cell proliferation, migration, and angiogenesis, which are crucial processes in cancer progression [10]. However, the potential mechanism of RPN1 on cancer cell progression is still unknown.

The PI3K/AKT/mTOR signaling pathway is a crucial cellular pathway involved in regulating various cellular processes, including cell growth, survival, proliferation, metabolism, and protein synthesis [13, 14]. It plays an important role in normal cellular function as well as in the development and progression of numerous diseases such cancer [15, 16]. Genetic alterations, such as activating mutations in PI3K, loss-of-function mutations in PTEN, or amplification/overexpression of AKT or mTOR, can lead to uncontrolled activation of the pathway, thereby promoting cell growth, proliferation, and survival [17–19]. Consequently, targeting this pathway has become a promising therapeutic strategy for certain cancers, and several drugs that inhibit this pathway have been developed and tested in clinical trials.

In this study, we investigated the expression of RPN1 in breast cancer tissue and the role of RPN1 in proliferation, migration, and invasion of breast cancer cells. We found that RPN1 expression was up-regulated in breast cancer tissues and cells. Further, RPN1 knockdown inhibited the proliferation, migration, and invasion of breast cancer cells. In addition, we examined the mechanism of RPN1 in the pathological development of breast cancer.

## Materials and methods

### Cells and transfection

Human normal breast tissue cell line MCF10A (American Type Culture Collection, Manassas, VA) and human breast cancer cell line MCF7 (American Type Culture Collection, Manassas, VA) were used in this study. MCF10A cells and MCF7 cells were cultured in DMEM/Ham’s F-12 (GIBCO-Invitrogen, Carlsbad, CA) medium and DMEM (GIBCO-Invitrogen, Carlsbad, CA) medium, respectively. Cells were incubated at 37 ℃ in an incubator with 95% air and 5% CO_2_. All cells used in experiments were in the linear phase of growth. Before transfection experiments, the cells were seeded in 6 well plates and allowed to grow to 50–60% confluence. To generate RPN1 knockdown cell models, RPN1 shRNA lentiviral particles (sc-36,420-V, Santa Cruz) or control shRNA lentiviral particles (sc108060, Santa Cruz) were transfected into MCF7 cells according to the manufacturer’s instructions. After 24 h of transfection, the cells were used for the next experiment. RPN1 expression was examined by western blotting.

### Human tissues

Totally 110 breast cancer patients were included in this study. All patients received cancer therapy at the Changzhou Wujin People’s Hospital between February 2021 and November 2022. The study protocols were approved by the Institutional Review Board of Changzhou Wujin People’s Hospital, and the study was conducted in accordance with the Declaration of Helsinki. All patients provided written informed consent to participate in the study.

### RNA isolation and real-time quantitative PCR (qPCR)

Total RNA was extracted from cell samples or human tissues using TRIZOL reagent (Invitrogen, 15596-026) and a RNeasy Mini kit (Qiagen, Valencia, CA, USA) according to the manufacturer’s instructions. The RNA concentration and optical density (OD) ratios at 260 and 280 nm were measured by NanoDrop (Thermo Fisher Scientific, USA). QRT-PCR was conducted using a one-step SYBR PrimeScript plus RT-PCR kit (Takara, HRR096A) by ABI 700 Real-Time PCR Detection system (Thermo Fisher Scientific, USA). Glyceraldehyde-3-phosphate dehydrogenase (GAPDH) was used as an internal control for the measurement of target mRNA expression levels .

The stem loop sequence for mRNA reverse transcription is 5′-GTCGTATCCAGTGCGTGTCGTGGAGTCGGCAATTGCACTGGATACGAC-3′. qPCR primer sequences are as follows: universal reserve primer, 5′- CAGTGCGTGTCGTGGAGT-3′; RPN1, 5′-TTGGAGAGCTGAGGTCATT-3′ (forward) and 5′-ATACACACACCAAACATCCATA-3’ (reverse); GAPDH, 5′-AGTCAGCCGCATCTTCTT-3′ (forward) and 5’-GCCCAATACGACCAAATCC-3′ (reverse). The relative mRNA expression levels were calculated using the 2^−∆∆Ct^ method [20, 21].

### Cell viability assay

A cell counting kit-8 (CCK-8) (Dojindo Laboratories, Kumamoto, Japan) was used to measure the viability of cells. The cells were seeded at a density of 1 × 10^4^ cells per well in 100 µL of medium into 96-well plates and cultured overnight. On the next day, the cells were treated with 740Y-P (20 µM) [20, 21] and incubated for 24 h in five parallel wells. To measure cell viability, 10 µL of CCK-8 solution was added to each well for another 2-h incubation. The absorbance was measured at a wavelength of 450 nm using a microplate reader (Bio-Rad Laboratories, Richmond, CA, USA). Cell viability was expressed as a percentage of that of the control (untreated) cells.

### Flow cytometry

For apoptosis analysis, an Annexin V-FITC/propidium iodide (PI) apoptosis kit (4 A Biotech, Beijing, China) was used under the manufacturer’s instructions. Control or PRN1 knockdown MCF7 cells were harvested, washed with PBS, and re-suspended in 100 µL binding buffer (2 × 10^5^ cells). After an incubation with 5 µL Annexin V–FITC for 5 min at room temperature in dark, 5 µL PI and 400 µL binding buffer were added to the cells for a 20-min incubation at room temperature in dark. The stained cells were examined by flow cytometer as instructed by its manufacturer. The data were analyzed with a flowjo software. Annexin V positive indicates that cells are in early apoptosis, while both FITC Annexin V and PI positive in late apoptosis or already dead [20, 21].

### Wound healing assay

MCF7 cells were seeded into 6-well plates and cultured to 70–80% of cell confluence. Upon a 24-h treatment with fetal bovine serum (FBS)-free medium, straight wounds were made using a 200 µL sterile tip in the center of the monolayer cells. After washing with medium to remove non-adherent cells, the wounded monolayers were treated with or without 20 µM 740Y-P, and images of the wound gaps were obtained under an Olympus BX61 microscope (Tokyo, Japan) at 0 and 24 h. The wound areas were quantitatively evaluated using Image J (NIH, USA) software. The migration rate (%) = (scratch width at 0 h − scratch width at 24 h/scratch width at 0 h) × 100%. To reduce variability in the results, multiple views of each well were documented, and each group experiment was repeated at least three times [20, 21].

### Transwell invasion assay

Cell invasion assay was performed in a transwell chamber with a polyethylene terephthalate filter membrane containing 8.0 μm pores on 24-well plates (Corning, USA). The filter membranes were coated with Matrigel. Specifically speaking, the cells (1 × 10^5^ cells/mL) suspended in 200 µL of serum-free medium were seeded into the upper transwell chamber with or without 740Y-P. The lower chamber was filled with medium containing 10% FBS. Following 24 h of culture, uninvaded cells were completely wiped with a cotton swab. The invading cells on the underside of the membrane were fixed with formaldehyde (4%) and stained with crystal violet (0.1%). The invading cells were then visualized by a fluorescent microscope (Olympus, Japan) [20, 21].

### Western blotting analysis

Total cellular protein was lysed using the RIPA buffer (Cell Signal Technology, Inc., MA, USA) and quantified by a BCA kit (Beyotime Biotechnology, Shanghai, China) following the principle of manufacturers’ protocols. Then 20 µg of the total protein was separated with 10% SDS-PAGE and then transferred to a PVDF membrane (Millipore, Billerica, MA). The membrane was blocked by pre-incubation with 5% skim milk for 30 min at room temperature and then incubated with the primary antibodies, including RPN1 (12894-1-AP, 1:500, Proteintech), PI3K (4292, 1:1,000, Cell Signaling), AKT (ab179463, 1:2,000, Abcam), mTOR (ab2732, 1:3000, Abcam), p-PI3K (ab182651, 1:500, Abcam), p-AKT (9271, 1:1,000, Cell Signaling), p-mTOR (2974, 1:1,000, Cell Signaling) and GAPDH (#2118, 1:1,000, Cell Signaling) at 4 °C overnight. On the next day, after three washes, the membranes were incubated with corresponding secondary antibodies, including goat anti-mouse IgG-HRP (sc-2005, Santa Cruz) and goat anti-rabbit IgG-HRP (sc-2004, Santa Cruz) diluted at 1:5,000 for 30 min at room temperature. The target protein was detected using Pierce™ ECL Western (Thermo Scientific, 32,209) and photographed by the chemiluminescence imaging analysis system (Bio-Rad, USA). The relative gray value was quantified by Image J (NIH, USA). GAPDH was used as the internal control [20, 21].

### Statistical analysis

Statistical analysis was performed using GraphPad Prism 8.0.2. Data were shown as mean ± standard error of the mean (SEM). The Student’s t-test was used to evaluate the differences between the control group and the experimental group, and one-way analysis of variance followed by Tukey’s test to evaluate the differences among multiple groups. *P* < 0.05 was considered statistically significant.

## Results

### RPN1 is notably up-regulated in breast cancer tissues and cells

**Fig. 1:**
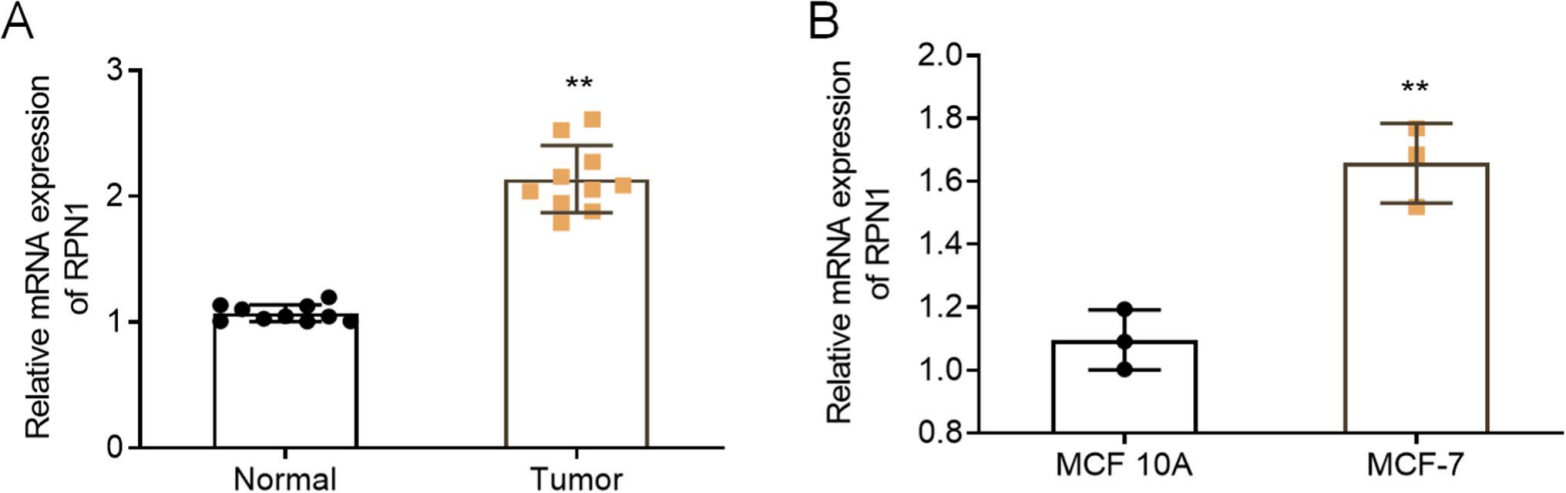
RPN1 mRNA expression level is increased in breast cancer tissue/cells. **A** qRT-PCR assay for RPN1 mRNA expression in 25 paired breast cancer tissues and adjacent non-tumor tissue samples. **B** qRT-PCR assay for RPN1 mRNA expression in breast cancer cell line MCF7 and normal human breast epithelial cell line MCF10A. * * *P* < 0.05

First, we performed qRT-PCR to monitor the RPN1 mRNA expression level in normal breast tissues and breast cancer tissues of patients. According to the qRT-PCR results, the RPN1 mRNA level was significantly increased in breast cancer tissues than that in normal breast tissues (*n* = 10, *P* < 0.05, Fig. 1A). We also compared the RPN1 mRNA expression level in normal breast mammary epithelial cell line MCF10A and breast cancer cell line MCF7. As shown in Fig. 1B, similar to Fig. 1A, the RPN1 mRNA expression was significantly up-regulated in MCF7 cells (*P* < 0.05). These results suggest that RPN1 may be related to pathogenesis of breast cancer.

### The effect of RPN1 on the PI3K/AKT/mTOR signaling axis in MCF7 cells

**Fig. 2:**
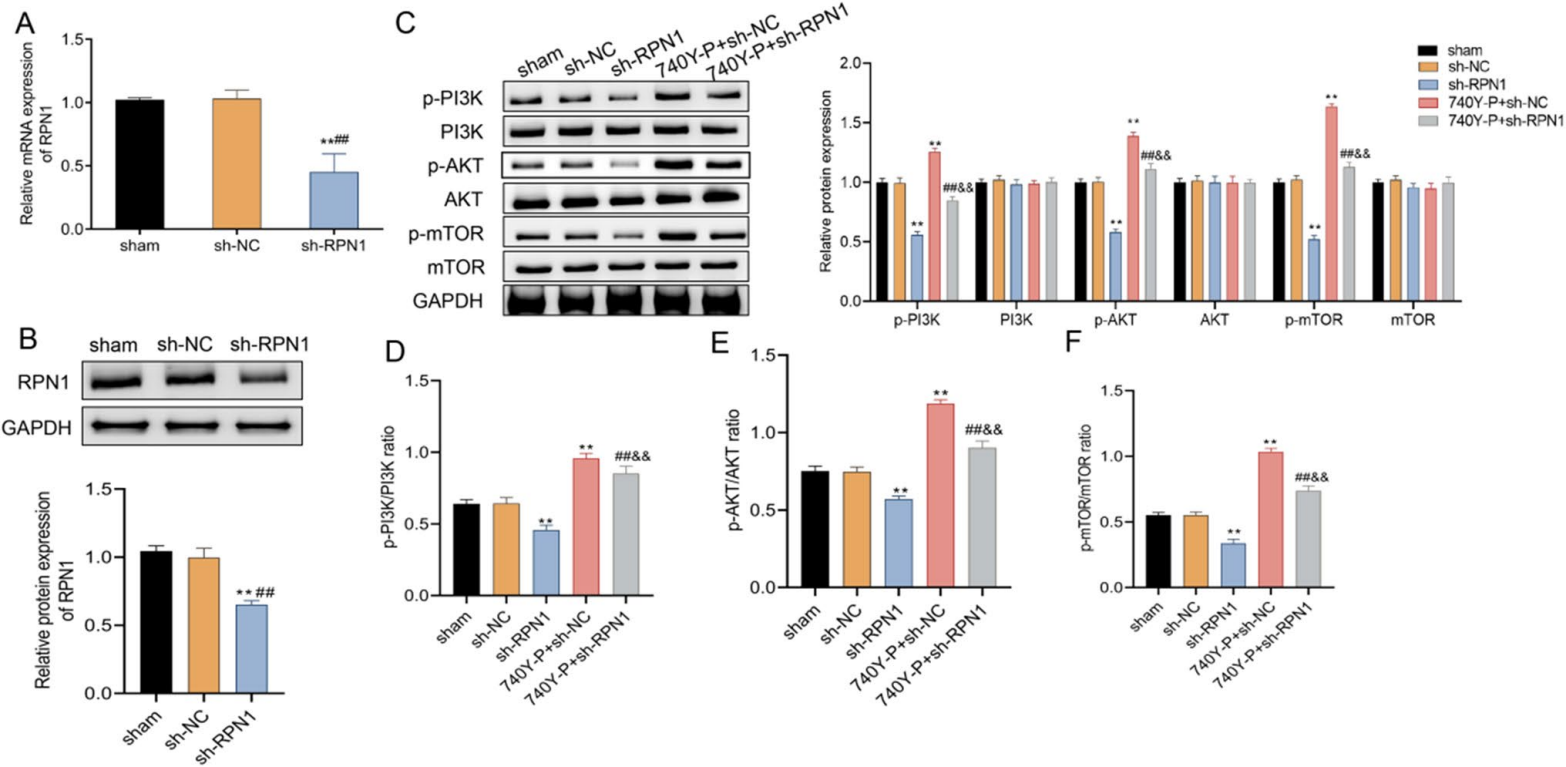
The effect of RPN1 knockdown on the PI3K/AKT/mTOR signaling axis. **A** qRT-PCR assay for the measurement of RPN1 mRNA expression in shRNA RPN1 transfected cells. **B** Western blot for the measurement of RPN1 protein expression in shRNA RPN1 transfected cells. **C** Western blot for the measurement of the PI3K/AKT/mTOR and their phosphorylation expression. The ratios of p-PI3K/PI3K (**D**), p-AKT/AKT (**E**) and p-mTOR/mTOR (**F**) were statistically analyzed. ** *P* < 0.05 (vs. sh-NC group). ##&& *P* < 0.05 (vs. 740Y-P + sh-NC group)

The PI3K/AKT/mTOR signaling is a vital pathway in cancer cells that regulates cell growth, migration, and invasion in breast cancer [22, 23]. To investigate whether RPN1 functions in breast cancer pathogenesis through the activation of PI3K/AKT/mTOR signaling, we analyzed the impact of RPN1 on PI3K/AKT/mTOR signaling pathway activation. We first established RPN1 knockdown MCF7 cell models via transfection of shRNA RPN1 lentivirus and examined the levels of RPN1 mRNA and protein. As shown in Fig. 2A, RPN1 mRNA expression level was significantly decreased in shRNA RPN1 transfected cells compare to controls. Similarly, RPN1 protein expression in shRNA RPN1 transfected cells was markedly down-regulated (Fig. 2B). To examine the effect of RPN1 on PI3K/AKT/mTOR signaling axis, the transfected MCF7 cells were treated with 740Y-P, a PI3K activator. After 24 h, the levels of p-PI3K, p-AKT, and p-mTOR in RPN1 shRNA transfected MCF7 cells in the absence and presence of 740Y-P were all elevated. The results showed that RPN1 knockdown lowered the levels of p-PI3K, p-AKT, and p-mTOR compared with control shRNA transfected cells (sh-NC) (Fig. 2C). Meanwhile, 740Y-P treatment elevated the levels of p-PI3K/PI3K, p-AKT/AKT, and p-mTOR/mTOR in the control shRNA transfected cells (sh-NC), while RPN1 knockdown dramatically decreased the levels of p-PI3K/PI3K, p-AKT/AKT, and p-mTOR/mTOR in the RPN1 shRNA transfected cells (sh-RPN1) (Fig. 2C–F). Taken together, RPN1 may play a role in MCF7 cells via activating the PI3K/AKT/mTOR signaling pathway.

### The effect of RPN1 on MCF7 cell proliferation and apoptosis

**Fig. 3:**
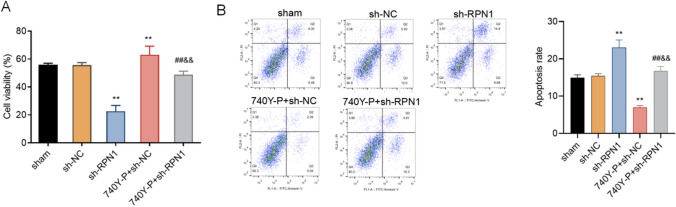
RPN1 promotes the proliferation of breast cancer cells via the PI3K/AKT/mTOR signaling pathway. **A** Cell availability assay in sh-NC or sh-RPN1 transfected MCF7 cells with or without treatment of 740Y-P. **B** Apoptosis analyzed by flow cytometry in sh-NC or sh-RPN1 transfected MCF7 cells with or without treatment of 740Y-P. **P < 0.05 (vs. sh-NC group), ##&& *P* > 0.05 (vs. 740Y-P + sh-NC group)

Then we examined the effect of RPN1 on MCF7 cell viability by carrying out the experiments of proliferation and apoptosis. As shown in Fig. 3A, RPN1 knockdown led to a decline in the viability in the sh-RPN1 group compared with the sham group and the sh-NC group. 740Y-P treatment significantly reduced the cell proliferation level in the 740Y-P + sh-RPN1 group compared with the 740Y-P + sh-NC group. RPN1 knockdown has been demonstrated to induce cell apoptosis triggered by ER stress [10]. Subsequently, we examined the effect of RPN1 on cell apoptosis by flow cytometry. Our results showed that compared with the sh-NC group, the apoptosis of the sh-RPN1 group was significantly increased, and 740Y-P treatment significantly decreased the apoptosis of the sh-NC group. On the other hand, 740Y-P treatment recovered apoptosis in sh-RPN1 transfected cells compared with the sh-NC group with treatment of 740Y-P (Fig. 3B).

### The effect of RPN1 on migration and invasion of MCF7 cells

**Fig. 4:**
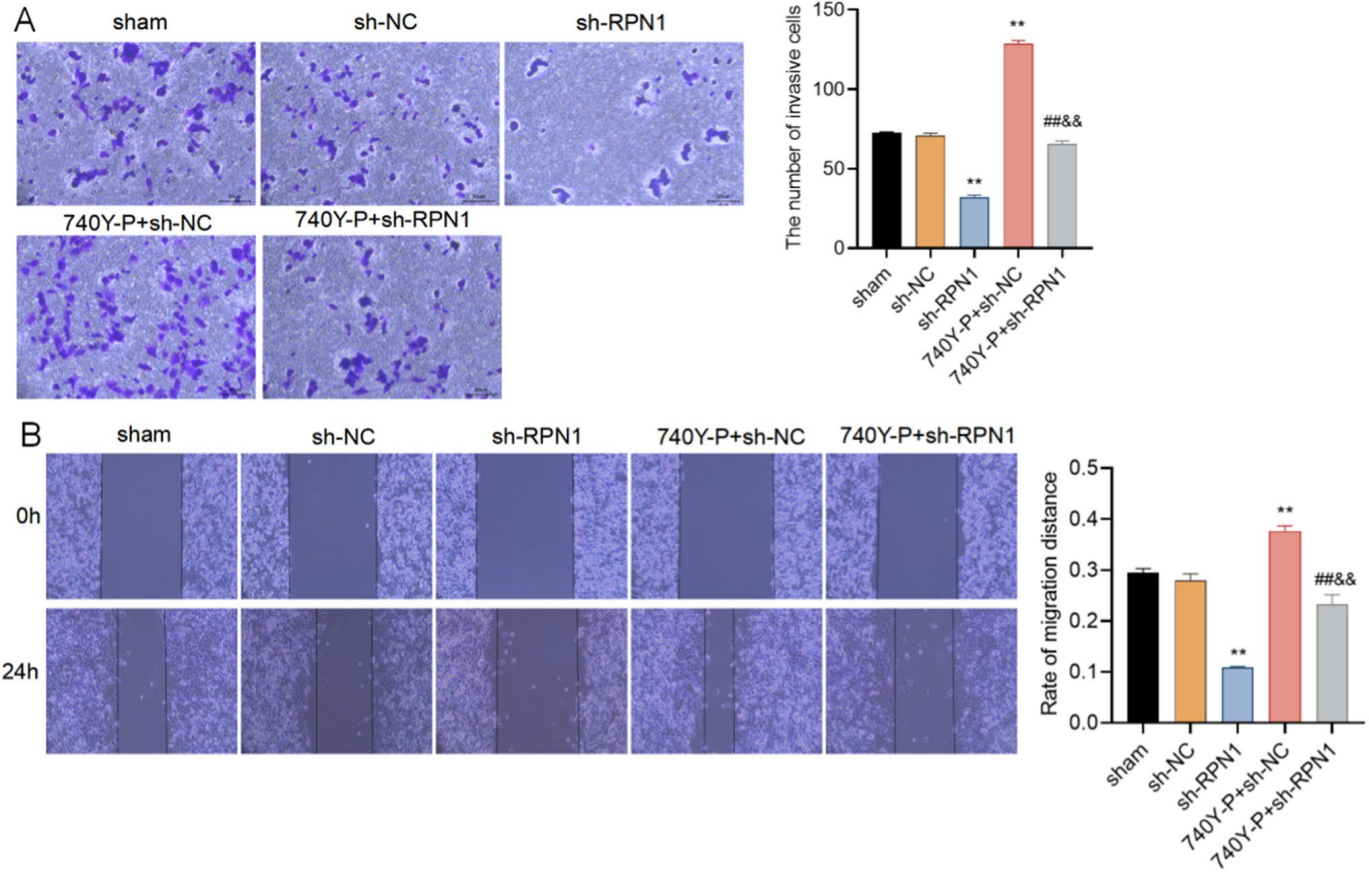
RPN1 promotes migration and invasion of breast cancer cells via the PI3K/AKT/mTOR signaling pathway. **A** Transwell assay in sh-NC or sh-RPN1 transfected MCF7 cells with or without treatment of 740Y-P, a PI3K activator. **B** Wound healing assay in sh-NC or sh-RPN1 transfected MCF7 cells with or without treatment of 740Y-P. **P < 0.05 (vs. sh-NC group), ##&& *P* > 0.05 (vs. 740Y-P + sh-NC group)

To explore whether RPN1 has an impact on MCF7 cell migration and invasion, we performed wound healing assay and transwell invasion assay. As shown in Fig. 4A, RPN1 knockdown significantly inhibited the number of invasive cells. 740Y-P, a PI3K activator, induced invasion of sh-NC transfected MCF7 cells. However, 740Y-P treatment could effectively reduce invasion in RPN1 knockdown cells compared with sh-NC transfected MCF7 cells (Fig. 4A), suggesting that RPN1 functioned via modulating the PI3K/AKT/mTOR pathway. Similar results were also observed in the wound healing assay (Fig. 4B). These results suggest that RPN1 could induce the migration and invasion of MCF7 cells via activating the PI3K/AKT/mTOR signaling pathway.

## Discussion

In the present study, we found that RPN1 was highly expressed in breast cancer tissues than it was in adjacent non-tumor tissues. Compared with the normal human breast epithelial cell line MCF10A, there was also an increased tendency of RPN1 expression in breast cancer cell line MCF7. RPN1 knockdown attenuated the levels of p-PI3K/PI3K, p-AKT/AKT, and p-mTOR/mTOR, proliferation, migration, and invasion while increasing the apoptosis of MCF7 cells. Our findings revealed that RPN1 functioned via the activation of the PI3K/AKT/mTOR signaling pathway.

Ribophorins, including RPN1 and RPN2, are highly-conserved glycoproteins located exclusively in the membranes of the rough ER [12, 24]. RPN1 is a protein involved in the process of protein synthesis within cells and is responsible for N-glycosylation in the ER of cells [25]. Abnormal or altered N-glycosylation have been suggested to influence the signaling pathways involved in cell proliferation, migration, and angiogenesis in cancer progression [26, 27]. Accumulating studies have reported that RPN2 is implicated in multiple cancers, such as breast cancer [28], small-cell lung cancer [29], bladder cancer [30], and colon carcinoma [31]. However, scant research has been done on the effect of RPN1 on cancers. Considering that RPN1 and RPN2 belong to the same family of proteins, and that RPN2 functions in various tumors, we therefore hypothesized that RPN1 might be a carcinogenic gene in breast cancer. Our study results showed that RPN1 was overexpressed in breast cancer tissues and cells. Subsequent assays uncovered that knockdown of RPN1 not only inhibited the proliferation, migration, and invasion of MCF7 cells in vitro, but also induced apoptosis of MCF7 cells. These findings align with those of Ding et al. [10]. Our study indicated that RPN1 played a vital role in the pathophysiology of breast cancer.

Constitutive activation of the PI3K/AKT/mTOR signaling pathway has been found in multiple human cancers, including breast cancer [32], ovarian cancer [33], and colorectal cancer [34]. This signaling pathway is essential in regulating tumor cell growth, metastasis, and apoptosis [35–37]. Han et al. reported that RPN2 promoted the growth and metastasis of bladder cancer by activating the PI3K/AKT/mTOR signaling pathway [30]. Therefore, we investigated how RPN1 affects the PI3K/AKT/mTOR signaling pathway in breast cancer. As expected, RPN1 knockdown induced the downregulation of p-PI3K, p-AKT, and p-mTOR in MCF7 cells and the increase of cell apoptosis. We further analyzed the involvement of RPN1 in the PI3K/AKT/mTOR signaling pathway using the PI3K activator, 740Y-P. It was discovered that 740Y-P treatment significantly increased proliferation, migration, and invasion but triggered apoptosis in sh-NC transfected MCF7 cells. On the contrary, knockdown of RPN1 notably attenuated these levels even after the treatment of 740Y-P. These data suggested that RPN1 may function via the PI3K/AKT/mTOR signaling pathway in breast cancer.

In this study, we revealed that RPN1 promoted the proliferation and invasion of breast cancer cells by activating the PI3K/AKT/mTOR signaling pathway. However, this study is subject to certain limitations. Firstly, only one breast cancer cell line (MCF7) was used in this study. Other breast cancer cell lines should be examined to confirm the expression and effect of RPN1. Secondly, we did not explore the possibility of RPN1 to work as a diagnostic indicator for breast cancer. Finally, although our results suggested that RPN1 promoted the proliferation and invasion of breast cancer cells by activating the PI3K/AKT/mTOR signaling pathway, the main target of RPN1 and the specific downstream signaling pathway of PI3K/AKT/mTOR require additional investigation.

## Conclusion

In summary, RPN1 is clinically overexpressed in breast cancer tissues of patients. Biologically, our in vitro experiments clearly confirmed the high expression of RPN1 in the breast cancer cell line MCF7. Furthermore, the proliferation, migration, and invasion of MCF7 cells are significantly inhibited after RPN1 knockdown. From the perspective of mechanism of action, RPN1 may act by activating the PI3K/AKT/mTOR signaling pathway. Although the detailed molecular mechanism is still unclear, it is apparent that RPN1 plays an important role in breast cancer and may be a potential therapeutic target for breast cancer.

## Data Availability

Data used in this study are available from the corresponding author upon reasonable request.
